# Anatomical correlates of blepharospasm

**DOI:** 10.1186/2047-9158-1-12

**Published:** 2012-06-15

**Authors:** Silvina G Horovitz, Anastasia Ford, Muslimah Ali Najee-ullah, John L Ostuni, Mark Hallett

**Affiliations:** 1 Human Motor Control Section, Medical Neurology Branch, National Institute of Neurological Disorders and Stroke, 10 Center Drive, Bdg10/7D37, Bethesda, MD, USA; 2 Brain Rehabilitation Research Center, Malcom Randall VA Medical Center, Gainesville, FL, USA; 3 Department of Psychology, University of Florida, Gainesville, FL, USA; 4 Office of the Clinical Director, National Institute of Neurological Disorders and Stroke, Bethesda, MD, USA

**Keywords:** Blepharospasm, Dystonia, Volumetric MRI, Magnetic resonance imaging, Diffusion weighted imaging

## Abstract

**Background:**

Focal dystonia is a neurological disorder characterized by unwanted muscle spasms. Blepharospasm is a focal dystonia producing an involuntary closure of the eyelid. Its etiology is unknown.

**Objective:**

To investigate if there are structural changes in the white and grey matter of blepharospasm patients, and if the changes are related to disease features.

**Methods:**

T1 and diffusion-weighted magnetic resonance imaging scans were collected from 14 female blepharospasm patients and 14 healthy matched controls. Grey matter volumes, fractional anisotropy (FA), and mean diffusivity maps were compared between the groups. Based on grey matter differences within the facial portion of the primary motor cortex, the corticobulbar tract was traced and compared between groups.

**Results:**

Changes in grey matter in patients included the facial portion of the sensorimotor area and anterior cingulate gyrus. These changes did not correlate with disease duration. Corticobulbar tract volume and peak tract connectivity were decreased in patients compared with controls. There were no significant differences in FA or mean diffusivity between groups.

**Conclusions:**

Grey matter changes within the primary sensorimotor and the anterior cingulate cortices in blepharospasm patients may help explain involuntary eyelid closure and the abnormal sensations often reported in this condition.

## Background

Blepharospasm is a form of focal dystonia characterized by involuntary closure of the eyelids, more common in women [1]. Although much effort has been dedicated to identifying its underlying causes, the full pathophysiology is still not established. Although blepharospasm is thought to be a basal ganglia disorder, no known histopathology associated with this disorder has been found.

Previous imaging studies investigating neural correlates of blepharospasm using voxel-based morphometry (VBM) implicated grey matter increases [2] or decreases [3] in the putamen in the patient group. Recently, changes in the grey matter of sensorimotor area were observed in blepharospasm patients [4,5]. Abnormalities in sensorimotor areas were seen in functional imaging studies of other primary focal dystonias, specifically, focal hand dystonia [6,7] and oromandibular dystonia [8]. Diffusion-weighted MRI studies (DW-MRI) found changes in the FA of the subgyral white matter of the sensorimotor cortex in DYT1 gene mutation carriers [9]; white matter changes were observed in the corticobulbar/corticospinal tract in spasmodic dysphonia patients[10]. However, to date, no white matter differences were found in patients with blepharospasm [11]. Nonetheless, tracer studies in macaques [12], human functional neuroimaging [13], and transcranial magnetic stimulation studies[14] suggest that the primary motor cortex, as well as the cingulate cortex, may be involved in blepharospasm pathophysiology.

The present study combines VBM and DW-MRI techniques to examine anatomical correlates of blepharospasm restricted to female blepharospasm patients to make the group more homogeneous.

## Participants and methods

The Institutional Review Board of the National Institutes of Health approved the experiment, and all participants gave their written informed consent. We collected structural and diffusion data from 14 female blepharospasm patients (Table 1) (59.9 ± 6.1 years) and 14 age- and handedness-matched female healthy volunteers (58.5 ± 5.6 years) utilizing a 3T GE Excite scanner using an 8-channel receiver only coil (General Electric Medical System, Milwaukee, WI, USA). 3D T1-weighted scans were collected using magnetization-prepared rapid acquisition gradient echo (MPRAGE) sequence (TR = 10 ms, TI = 450ms, TE = minimum full (3ms), Flip Angle = 10 degrees, Bandwidth = 31.25, FOV = 240 mm, phase FOV = 192 mm, matrix = 256 x 256, and 128 axial locations, 9 min 13 sec.). Diffusion-weighted data were acquired using TE/TR = 73.4/13000 ms, FOV = 240x240 mm^2^; matrix = 96x96 zero-filled to 256x256; 54 contiguous axial slices with slice thickness of 2.4 mm; 33 non-co-linear gradient directions; b-value = 1000 s/mm^2^; 3 b-zero volumes). We repeated this sequence twice to improve the signal-to-noise ratio.

**Table 1:** Characteristics of Blepharospasm Patients

**Patient ID**	**Handedness**	**BFM score (eye portion)**	**Age**	**Disease duration** **(years since diagnosis)**	**Years in Btx treatment**
1	RH	4	65	9	9
2	RH	8	65	6	5
3	RH	4	51	3	none
4	RH	8	60	6	0
5	RH	4.5	58	10	9
6	RH	4	59	4	4
7	RH	8	66	13	13
8	RH	4	60	1	1
9	RH	8	50	10	10
10	LH	6	59	8	8
11	RH	4	64	10	10
12	RH	4	69	24	24
13	RH	6	63	2	2
14	RH	6	50	9	3

To perform echo planar imaging (EPI) distortion correction, T2-weighted images were acquired (FSE-T2: TE/TR = 120/5100 ms; matrix = 256x256) using the slice prescription of the diffusion-weighted dataset.

### VBM analysis

We used the FSL-VBM software (http://www.fmrib.ox.ac.uk/fsl), which implements VBM style analysis [15,16]. Pre-processing included: image skull-stripping; tissue type segmentation [17,18]; registration of the grey matter partial volume images to a standard space; and non-linear transformation to create a study-specific template [19-22] with a resolution of 2x2x2 mm. Original grey matter images were registered to the study template. To correct for local expansion or contraction produced by the registration grey matter volume, images were modulated by dividing by the Jacobian of the warp field. The modulated registered images were then smoothed using an isotropic Gaussian kernel with sigma of 3 mm resulting in 6.9 mm smoothing. We performed a two-tailed *t*-test to find differences between groups. Significance was set at p = 0.01, with cluster size =80 voxels. For the regions of significant decreases, we correlated the patients’ grey matter densities with their disease durations, Burke-Fahn-Marsden scores, and ages.

### Analysis of diffusion images

Using the TORTOISE software (http://www.tortoisedti.org), diffusion-weighted data were corrected for motion, eddy currents, and EPI distortions prior to nonlinear tensor fitting [23]. The FA and directionally encoded color (DEC) maps were visually examined to ensure proper fit and absence of obvious artifacts caused by uncorrected image distortions [24]. We carried out voxel-wise statistical analysis of the FA maps using FSL’s [25] TBSS (Tract-Based Spatial Statistics [26]). FA maps were registered in standard space using nonlinear registration, which uses a b-spline representation of the registration warp field [20,21,27]. These maps were averaged to create a mean FA image, which was then thinned to produce a mean FA skeleton, representing centers of the tracts common to all subjects. The skeleton was binarized and served as a study-specific template. Values nearest to a given tract center skeleton were projected onto the skeleton from the standard space FA maps of each subject and compared between groups.

To further examine affected neural networks in blepharospasm patients, we analyzed the left corticobulbar tract (CBT), based on the morphometry results. We used regions showing differences in grey matter between patients and controls as origins for probabilistic tractography [28]. We registered masks of the left face portion of the precentral gyrus, identified in our VBM analysis, to subjects’ native diffusion space using the inverse of the nonlinear transform from the TBSS analysis [20,21]. In addition to the seed mask acquired from the VBM analysis, we used exclusion and waypoint masks, drawn in original native space, to guide the tracking algorithm. We used the first mid-sagittal slice of the right hemisphere as an exclusion mask to ensure that only the tracts from the left precentral gyrus were traced. We used a single slice in the mid-pons as a waypoint and termination mask for the CBT tracing. We registered the exclusion and termination masks to the re-sampled DW data using linear transform [19,22]. For the CBT, we computed the average volume and the connectivity index, represented by the number of probabilistic streamlines that originated in the seed region and reached the target.

The mask volume of the facial portion of precentral gyrus for the healthy volunteers was larger than that for the patients due to group differences in grey matter volume in this region (see Results). Therefore, we compared tract volumes and connectivity indices with mask volume to confirm that our results were not biased by mask size.

## Results

### VBM

**Figure 1:**
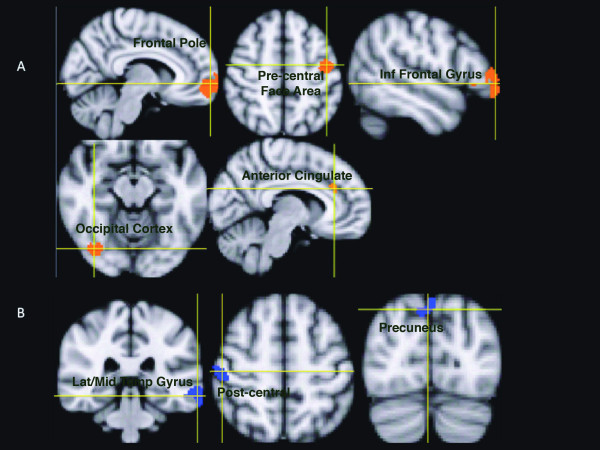
** Regions showing changes in grey matter volume between blepharospasm patients and healthy volunteers.A**) Decreases are shown in red; **B**) increases are shown in blue. See Table 2 for quantification.

**Figure 2:**
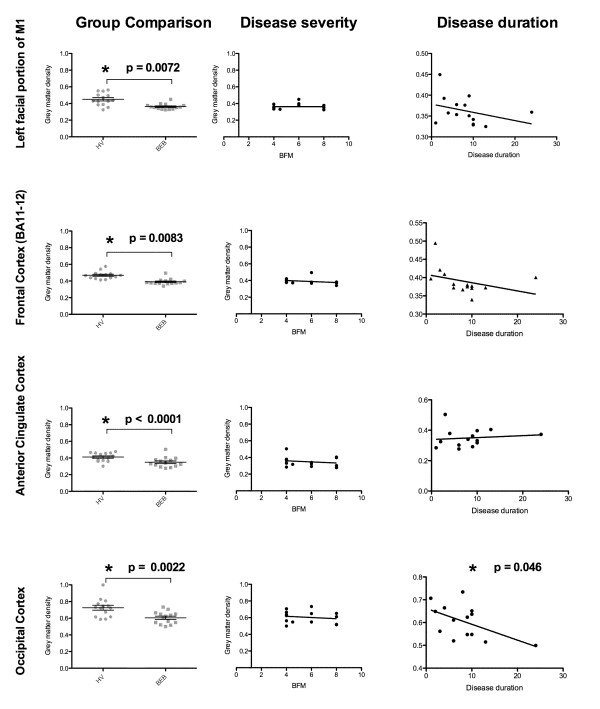
** Left: Mean value for grey matter density for the regions shown in Table **2**a.** * indicates significant difference (p < 0.01) between healthy volunteers and blepharospasm patients. **Center:** correlation of grey matter density with disease severity (BFM scores). **Right:** correlation of grey matter density with disease duration

Total grey and white matter volumes were not significantly different between groups (p > 0.6). Decreased regional grey matter volume in patients included the right orbitofrontal cortex (frontal pole), left facial portion of the precentral cortex (primary motor cortex), left lateral inferior frontal gyrus, right occipital cortex, and right anterior cingulate gyrus. The grey matter volumes of the left lateral middle temporal gyrus, right postcentral gyrus, and bilateral precuneus were increased in the blepharospasm patients compared with controls (Figure 1, Table 2). Disease duration only correlated with amount of grey matter decrease in the occipital cortex (Figure 2). Neither age nor Burke-Fahn-Marsden score correlated with the changes in these areas.

**Table 2:** Regions showing statistically significant grey matter volume differences between blepharospasm patients and healthy volunteers (p < 0.01*, p < 0.001**, uncorrected)

**Volume** (**mm**^**3**^)	**Maximum t-scores**	**MNI152 Coordinates (mm)**	**Structure**
a) grey matter volume in patients < healthy volunteers			
2090	4.90**	34, 62, 8	R Frontal Pole
604	4.09**	−40, -10, 38	L Precentral Gyrus (Face Portion)
490	4.36**	−52, 42, 8	L Lateral Frontal Pole/L Inferior Frontal Gyrus
228	3.62*	40, -74, -14	R Lateral Occipital Cortex
164	2.89*	12, 28, 34	R Anterior Cingulate Gyrus
b) grey matter volume in patients > healthy volunteers			
436	3.39*	−68, -34, -20	L Lateral Middle Temporal Gyrus
296	3.37*	50, -20, 62	R Postcentral Gyrus
214	4.37**	0, -82, 48	Precuneus

a) patients < healthy volunteers; b) patients > healthy volunteers.

### Diffusion images

The voxel-wise analysis of the FA and mean diffusivity maps carried out using TBSS did not identify any statistically significant differences between the groups.

**Figure 3:**
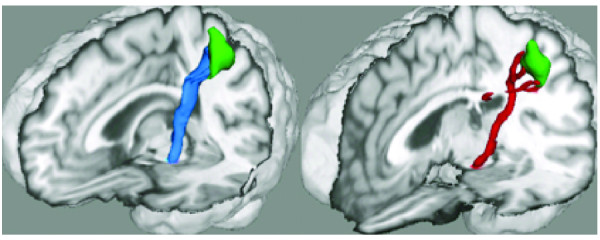
** Representative rendering of corticobulbar tract in a healthy volunteer (blue) and an age-matched blepharospasm patient (red).Right:** blepharospasm patient, **left:** age-matched healthy volunteer. Green: cluster showing significant decrease in grey matter volume in blepharospasm patient located in the facial portion of the precentral gyrus.

The average volume of the left CBT was significantly lower for patients (5503 ± 2082) than that for the healthy volunteers (7273 ± 2347), p-value = 0.02 (Figure 3). The average tract connectivity for patients (1304 ± 1518) was lower than that for the healthy volunteers (3355 ± 3878), p-value = 0.04.

The FA value distributions within the CBT fibers, adjusted for tract volume, did not significantly differ between the groups. There were no significant correlations between seed size and tract volume.

## Discussion

Our main findings are a significant increase within the right somatosensory cortex and a significant decrease in the facial portion of the left primary motor cortex and right anterior cingulate in blepharospasm patients grey matter when compared to healthy volunteers, in line with the concept of somatotopy of abnormality in dystonia [1]. These areas found to differ between groups are part of the “upper motor neuron” control of blinking. As the changes were unaffected by disease features such as duration, it is plausible they play a role as substrate in the etiology of the disorder. Results from our study could help targeting histopathological studies and, in turn, unravel the basic mechanisms of blepharospasm.

Originally believed to be a basal ganglia disorder [2,29], functional and structural differences in basal ganglia have been often elusive in neuroimaging studies [8,30] and were not present in our study. Cortical areas certainly seem critical. Our results highlight the presence of structural changes in the sensorimotor cortex and in the anterior cingulate of blepharospasm patients. In addition, reduction in the corticobulbar tract volume within the left hemisphere in blepharospasm patients may lead to decreased input from the primary motor cortex to the facial nuclei. The anterior cingulate is the primary locus of cortical input to the orbicularis oculi muscles controlling eyelid closure [12]. Inhibition of this medial frontal area using low-frequency repetitive stimulation results in electrophysiologic and clinical improvement in blepharospasm [31]. The cortical findings in these key areas related to the motor control of blinking reinforce the idea that TMS therapy could be optimized for the treatment of blepharospasm [32].

Our results show that grey matter near the central sulcus differs between groups in both hemispheres, but the identified changes in the two hemispheres differed. Patients have grey matter volume decrease in the left motor cortex, and grey matter volume increase in the right somatosensory cortex. It is possible that the pre- and post- central changes observed in this study are representations of the same pathological phenomena in the sensorimotor area. Misalignment of sulci could be responsible for seeing the effects as an increase of grey matter on one side of the sulcus or a decrease on the other; these are inherent limitations of the segmentation and registration procedures in VBM analysis.

Secondary findings in our study include a decrease in patients’ grey matter in the right orbitofrontal cortex, left inferior frontal cortex, left lateral side of the frontal pole and right lateral occipital cortex. The changes in the frontal lobes are in line with fMRI blink studies indicating these areas are involved in blink control [13,33,34]. Moreover, the inferior frontal cortex showed glucose metabolism abnormalities in blepharospasm patients [35]. Changes in the occipital area are difficult to interpret since this area is not related to blinking. However, the lateral occipital cortex had decreased activation during blinks [36], and our study indicates the changes are correlated to disease duration, suggesting these changes might be a consequence of the chronicity of the disorder.

Patients have increased grey matter in the left lateral middle temporal gyrus and bilateral precuneus, two areas that show increased activation during blink suppression [37].

As previously reported [11], blepharospasm patients did not differ from healthy volunteers in most of the diffusion parameters we explored. The TBSS method is based on the registration of a skeleton with the maxima FA value, and the FA values within the tract are similar between groups. In our study, the average volume and the connectivity of the tract became a more sensitive measure than the results from TBSS analysis. The decreases observed in the patients’ left CBT average volume and in the grey mater of the sensorimotor cortices could be indicative of the upper face motor dysfunction seen in blepharospasm.

The results of our study come solely from women. Although no earlier study in any of the focal dystonias identified a gender difference, it may well be that combining both genders increases the variance of the data. Medication and disease symptoms were not taken into account in this study. These factors are variable over time and it is unclear how they might affect the anatomy.

### Technical considerations

VBM analysis is sensitive to MR scan parameters due to partial volume effects inherent to imaging resolution. Directionality of changes in grey matter (increase or decrease) depends on the MR sequence used to acquire the data. Thus, the apparent discrepancies in directionality of changes in volume are due to acquisition parameters used in different studies. Our sample size is small, but the results are consistent with the literature. Furthermore, we believe that having a homogeneous population strengthen our results.

The parameters used for diffusion acquisition favors tractography in the superior-inferior direction, the main direction of the corticobulbar tract. These parameters are less sensitive to the lateral tracts, thus making it very difficult to follow fibers reliably from the anterior cingulate cortex. While results from diffusion data are a coarse representation of the complex neuronal networks of the brain, they provide a window into structural changes in the white matter, otherwise difficult to assess in vivo.

## Conclusions

We identified structural differences in the cortical areas responsible upper motor neuron control of blinking. The changes in the sensorimotor areas and in the anterior cingulate were not correlated with disease duration or severity, suggesting they might be fundamental to the disorder and not an effect of it. Anatomical changes within these regions may be responsible for abnormal upper facial muscle contractions seen in our blepharospasm population, although the exact mechanism is not yet known. Our findings contribute to a better understanding of the basic pathology in blepharospasm and suggest these areas could be targeted for treatment of blepharospasm.

## Abbreviations

DW-MRI, Diffusion-weighted MRI; TMS, Transcranial magnetic stimulation; VBM, Using voxel-based morphometry; EPI, Echo planar image; FA, Fractional anisotropy; DEC, Directionally encoded color; TBSS, Tract-Based Spatial Statistics; CBT, Corticobulbar tract.

## Competing interests

The authors declare that they have no competing interests.

## Authors’ contributions

AF carried out data analysis, interpretation and drafted the manuscript. MA-N carried out data acquisition and analysis. JLO assisted with data analysis. MH carried out study conception, design and interpretation of the data and wrote the manuscript. SGH carried out study design, data acquisition, analysis, and interpretation of the data and wrote the manuscript. All authors have given approval of the final version to of the manuscript.

The Intramural Research Program of the National Institute of Neurological Disorders and Stroke, National Institutes of Health supported this research.
